# Towards Understanding the Chemical Structure Modification of EVA Copolymer upon MAPLE Processing of Thin Films

**DOI:** 10.3390/ijms222111686

**Published:** 2021-10-28

**Authors:** Agata Niemczyk, Simona Brajnicov, Veronica Satulu, Jolanta Baranowska, Bogdana Mitu, Maria Dinescu

**Affiliations:** 1 Department of Materials Technology, Faculty of Mechanical Engineering and Mechatronics, West Pomeranian University of Technology, 19 Piastow Ave, 70-310 Szczecin, Poland; baranops@zut.edu.pl; 2 National Institute for Laser, Plasma and Radiation Physics, 077125 Bucharest, Romania; brajnicov.simona@inflpr.ro (S.B.); veronica.satulu@inflpr.ro (V.S.); maria.dinescu@inflpr.ro (M.D.)

**Keywords:** poly(ethylene-*co*-vinyl acetate), MAPLE, chemical structure

## Abstract

A series of coatings from poly(ethylene-*co*-vinyl acetate) (EVA) were obtained using the matrix-assisted pulsed laser evaporation (MAPLE) technique. By changing the process parameters, i.e., laser fluence and EVA co-polymer concentration in the target, coatings with various morphologies and topographies were produced. The evaluation of the film structure was based on an analysis of optical and atomic force microscopy and profilometry measurements. A detailed chemical structure investigation, conducted based on Fourier transform infrared (FTIR) and X-ray photoelectron spectroscopy (XPS) spectra, revealed that although the general structure was preserved, some alterations of ethylene (Et) and vinyl acetate (VAc) blocks took place. The most noticeable change was in the ester group that was transformed into ketone and carboxyl groups; nevertheless, some changes in the aliphatic main chain were also present. The chemical structure changes in EVA coatings took place regardless of the process parameters used. The use of chloroform as a solvent to dissolve the EVA copolymer was indicated as a possible reason of the changes as well as the tendency of EVA macromolecules to form clusters. Nevertheless, due to low level of structure alteration, it has been shown that the MAPLE technique can be successfully used to obtain coatings from polymers with more complex structures, which are soluble in a limited number of solvents.

## 1. Introduction

Including physical vapor deposition (PVD) methods in polymeric coating technologies has provided various advantages, which overcome some of the existing challenges of the traditional “wet coating” techniques [1,2,3]. For example, pulsed laser deposition (PLD) is a technique, which allows one to coat an arbitrarily selected material, resulting in a well-adhered thin polymer coating. Nevertheless, photochemical and photothermal degradation reactions, which accompany the process, represent a serious drawback, especially for applications in which a coating has to demonstrate the same (preserved) chemical structure as the polymer used as target. Additionally, it is already known that parts of macromolecules undergo chain scission during the laser ablation process (a higher molecular weight of polymer results in a higher percent of chain scission [4]), which can lead to a decreased molecular weight of deposited polymer chains or their crosslinking.

In terms of polymers synthesized by radical polymerization, especially homopolymers, scission and photodegradation processes occurring during ablation do not have such a negative impact on the final structure of the polymeric coating as for other types of polymers, mostly due to possible re-polymerization reactions during the deposition, either in the gas phase or on the surface. On the other hand, in the case of copolymers or polymers having such heteroatoms as, e.g., O, N, or S, multiple photoreactions and/or re-combination of the species created during ablation process can lead to a significantly altered (often degraded) structure of the polymer coating [5].

Poly(ethylene-*co*-vinyl acetate) (EVA) is a widely used copolymer, built from ethylene (Et) and vinyl acetate (VAc) blocks, varying in physical and chemical properties depending on the mass ratio of the Et and VAc [6,7,8]. Vinyl acetate block, a polar group, introduces the EVA copolymer amorphous phase and rubber-like character. It is responsible for certain properties such as flexibility, solubility in organic solvents and good adhesiveness [9]. However, the VAc group, consisting of two oxygen atoms, is the most vulnerable part of EVA macromolecules, quite easily undergoing photo- and thermo-degradation under the action of high energetic beams [10,11] as well as under the photon radiation during the PLD process [12].

A variation of PLD, in which, by using a frozen solution of a polymer, the photodegradative effect of the laser beam is significantly diminished, is the matrix-assisted pulsed laser evaporation (MAPLE) technique. During MAPLE, the majority of the laser energy is absorbed by the frozen solvent molecules, which, throughout their evaporation/ablation process, entrain polymer macromolecules and transfer them to the vapor phase, allowing for their deposition with a preserved chemical structure [13,14]. However, for such ideal results of unaltered transfer from the target to the substrate, certain requirements must be met. Firstly, the polymer material has to be highly soluble in the solvent and the solution should not be too viscous (typical solution concentration in MAPLE does not exceed 5 wt%). Secondly, the selected solvent has to absorb most of the laser beam, be highly volatile, not create a film under photochemical reaction, and not photodissociate to reactive species (which could react with transported polymer chains) [13,15].

In various studies, it has been defined that water is a solvent allowing the second part of the above-mentioned requirements to be fulfilled, giving a very good results in biomolecule depositions [16,17]; nonetheless, a significant number of polymers (especially synthetic ones) do not dissolve in water or are even sparingly soluble in organic solvents. Among them is also the EVA copolymer, which dissolves mostly in chlorinated or aromatic solvents, which are not recommended to be used in the MAPLE process [18], due to the possibility of forming highly reactive radicals. However, this does not mean that the MAPLE technique cannot be used to obtain polymer films with maintained chemical structures but points out that each type of polymer should be considered individually, with careful structural analysis of the deposit, to verify to what extent the chemical structure has been preserved.

The aim of our work was to develop coatings from EVA copolymer by the MAPLE technique using chloroform as the solvent and to verify the ability of the MAPLE method to preserve the original chemical structure of the copolymer. Defining and understanding the changes of chemical structures during the MAPLE process constitute an important element toward the more intensive utilization of this method for polymers with complex structures/copolymers and characterized by limited solubility. Creating an EVA coating of an appropriate quality using one of the PVD techniques, i.e., MAPLE, has not been reported so far. Obtaining such films opens a new and efficient way to produce multi-component elements for electronic devices such as sensors or organic–inorganic solar cells [19] as well as advanced materials in the biomedical field such as for drug delivery [8] or for the prevention of calcification in prosthetic cardiac valves [20,21].

Two series of coatings, obtained at different concentrations of EVA copolymer solutions and various laser fluences, have been compared analyzing the MAPLE process condition influence on the coating characteristic and possible changes in the copolymer chemical structure. The topography and morphology of the coating were evaluated qualitatively and quantitatively by optical and atomic force microscopies, and the chemical bonding was determined by a detailed analysis of infrared and X-ray photoelectron spectra.

## 2. Results and Discussion

Two series of coatings have been obtained, i.e., series with variable concentrations of the target EVA solution and series with variable laser fluence, all summarized in Table 1. To distinguish coatings obtained from a 1 wt% solution at 0.5 J/cm^2^ laser fluence, with different number of laser pulses, the one obtained with 36,000 pulses is marked with an asterisk (*).

**Figure 1:**
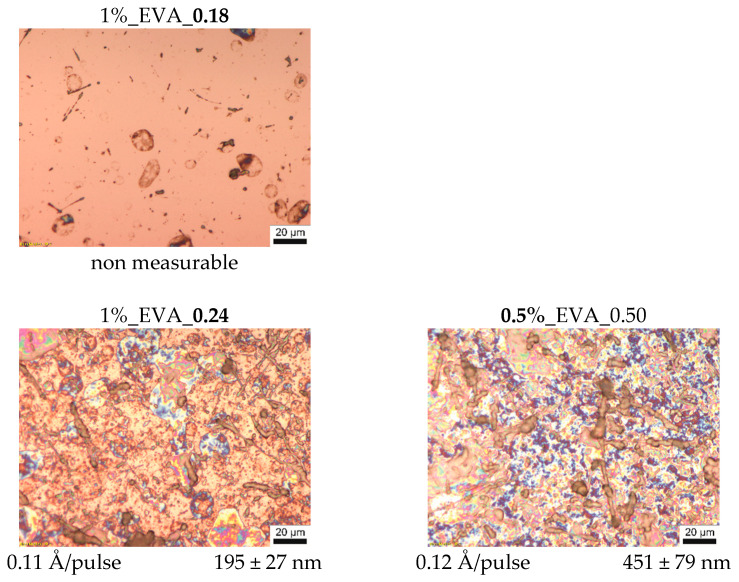

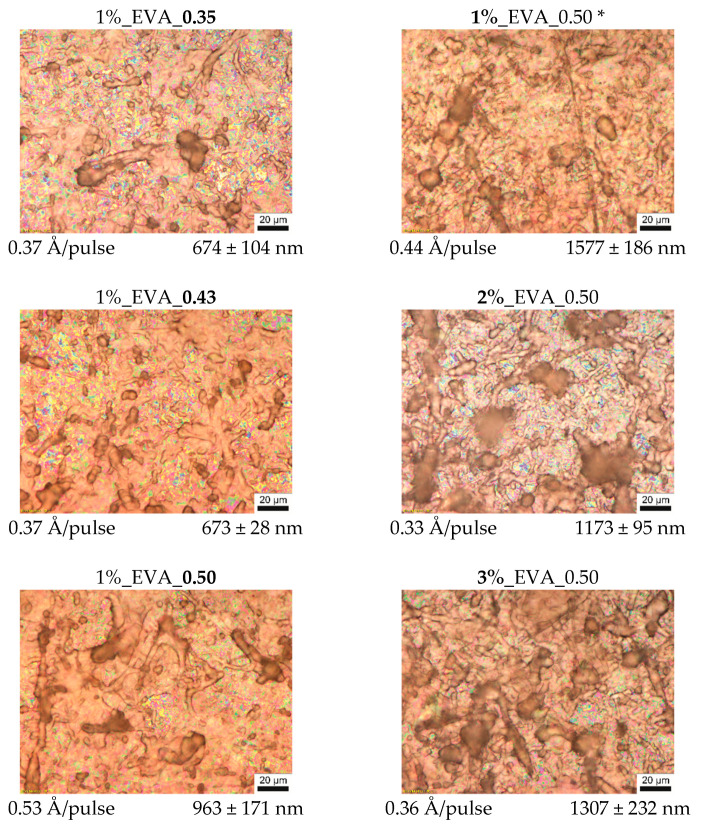
Optical micrographs of two series of EVA films with the values of process efficiency and coating thickness indicated for each sample.

Optical microscopy pictures with the values of thickness and deposition efficiency of the series of EVA coatings are presented in Figure 1. As can be noticed, the process conducted with 0.18 J/cm^2^ laser fluence does not lead to a continuous polymer film formation, indicating that this fluence is under the threshold deposition value.

With a laser fluence of 0.24 J/cm^2^ and upwards, continuous coatings are obtained. Their thicknesses and deposition efficiencies increase with an increasing fluence; however, the relationship between these two properties and the fluence is non-linear. This is due to the fact that the MAPLE is in a non-equilibrium and time-dependent process (the time dependency is also seen in some deviations from a linear correlation of thickness and number of pulses, which, in contrast, is characteristic of the PLD process). On the surface of EVA coatings obtained at higher fluences, i.e., 0.35–0.50 J/cm^2^ (Figure 1) a large number of polymeric particles with various shapes from circular to fiber-like ones was observed, and their presence can be explained as follows. The change of laser fluence mainly influences the ablated volume of the target and the amount of energy supplied to this part of material. However, the fluence value used during the MAPLE process is relatively low, and often it is below the threshold value to ablate the polymer macromolecules by themselves (from bulk target, without a solvent). Macromolecules are entrained and transported to the substrate by solvent molecules while they are passing into the gaseous state. Therefore, energy high enough to evaporate/ablate the solvent molecules must be supplied in the process. Nevertheless, if macromolecules are strongly entangled or they strongly interact with solvent molecules, polymer–solvent clusters will be ejected from the target since the macromolecules cannot be separated into individual chains during their transport towards the substrate. For solvent–polymer systems with a tendency to form clusters, an increased fluence will ensure a deposit with more clusters or clusters that are bigger in size [14,22], and this behavior is observed for the obtained series of EVA coatings at a higher laser fluence.

A similar tendency was observed for the EVA coating series obtained from the target solution with different concentrations at a fluence of 0.5 J/cm^2^, for which a higher concentration results in higher density of the polymeric (“bulky”) particles (Figure 1). A higher amount of the EVA copolymer macromolecules in the solution leads to a higher entanglement of the chains, which, in consequence, promotes the formation of the intricate structures, which are integrally ejected during ablation.

**Figure 2:**
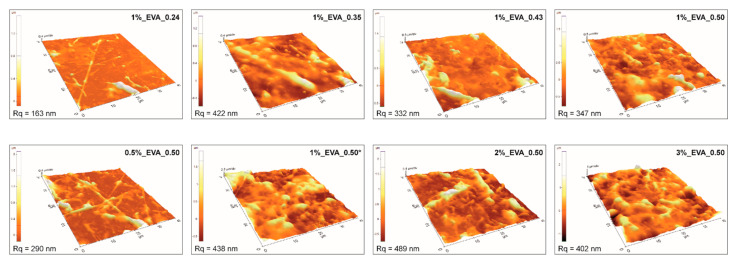
AFM images of two series of EVA coatings with root mean square roughness (R_q_).

Atomic force microscopy results (Figure 2) are consistent with the mentioned analysis, i.e., coatings obtained at a higher fluence or higher solution concentration have higher surface roughness. Consequently, the coatings obtained from 1%_EVA_0.24 and 0.5%_EVA_0.50 are characterized by the lowest surface roughness (R_q_), i.e., in a range of 200–300 nm. In general, moderate roughness is quite common for coatings obtained by the MAPLE technique, especially for polymers, which have a high level of inter- and intramolecular interactions, as a consequence of cluster formation.

**Figure 3:**
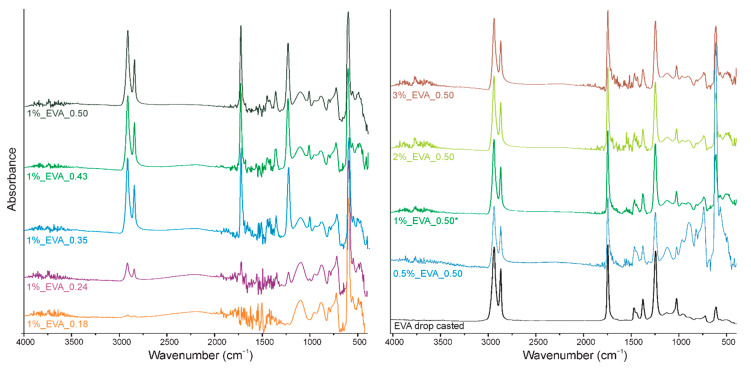
FTIR spectra of two series of EVA films: left with variable laser fluence and right with variable concentrations of the EVA solution.

**Figure 4:**
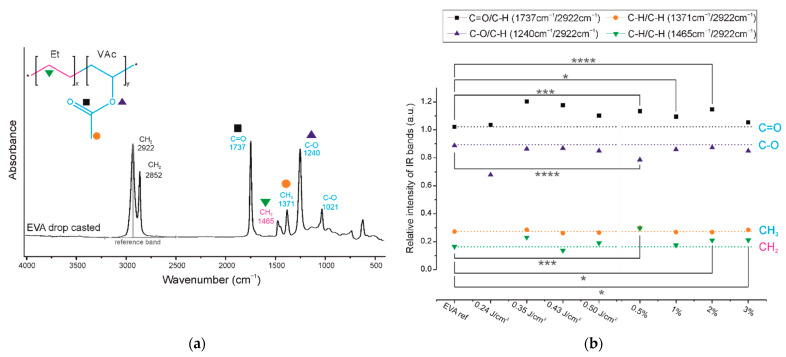
(**a**) FTIR spectrum of reference EVA coating with marked and assigned reference and characteristics. (**b**) Relative intensity of IR bans of two series of EVA films (statistical difference between reference and particular coating * *p* = 0.02, *** *p* = 0.0001, **** *p* < 0.0001).

The second part of this research is dedicated to a detailed analysis of the chemical structures of the coatings. The infrared spectrum of the EVA copolymer has several characteristic absorption bands (Figure 3 and Figure 4a), which can be assigned either to ethylene or to vinyl acetate block as follows: 1021 cm^−1^ and 1240 cm^−1^ are assigned to the C-O bond stretching of the VAc block, 1371 cm^−1^ is assigned to the C-H bond rocking of the VAc block, 1465 cm^−1^ is associated with the C-H bond bending or scissoring of the Et block, 1737 cm^−1^ is assigned to the C=O bond stretching of the VAc block, and 2852 and 2922 cm^−1^ are assigned to the C-H bond stretching of the EVA backbone chain.

In the spectra of all the obtained EVA coatings (Figure 3), regardless of the process conditions used, all characteristic absorption bands of the EVA copolymer are present, indicating that the copolymer did not undergo any substantial structural alteration during the MAPLE process. Nevertheless, a quantitative analysis of the IR spectra was conducted in order to evaluate the chemical structures of the EVA coatings more accurately. As such, for the spectrum of each coating, we selected a reference absorption band corresponding to the polymer backbone chain (at 2922 cm^−1^) and four characteristic bands (1240, 1371, 1465 and 1737 cm^−1^). By dividing the intensity of the particular characteristic band by the intensity of reference band, within the same spectrum, we obtained the so-called “relative intensity of characteristic bands”. These relative intensities for the two series of coatings are presented in Figure 4b in comparison with the relative intensities of the initial EVA copolymer. The quantified results confirm that the main chemical structure was retained; however, some deviations were noticed from the relative intensity of the characteristic bands of the reference coating (EVA ref). Thus, changes occurred in the amount of a particular bond. The deviations noticed for coatings 1%_EVA_0.24, 1%_EVA_0.35 and 0.5%_EVA_0.50, are associated with the low thicknesses of the coatings, resulting in the low intensity of the IR absorption bands, together with the high roughness of the surface coating which additionally caused the increased signal noise (Figure 1 and Figure 3).

**Figure 5:**
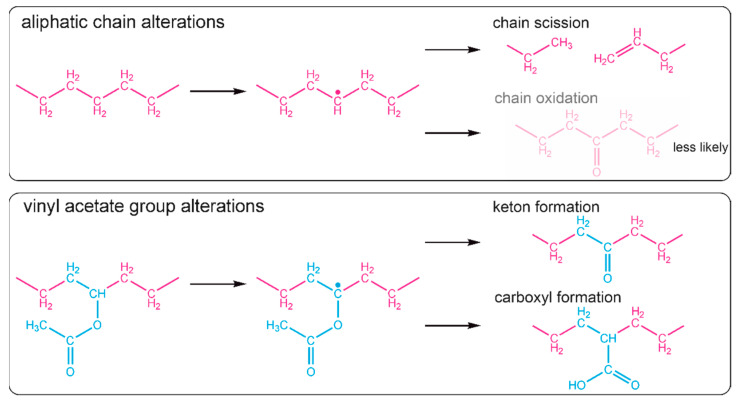
Possible reaction paths of Et or VAc blocks of EVA macromolecules during MAPLE process.

Small changes in the intensity of the band at 1465 cm^−1^ corresponding to CH_2_ groups of the long aliphatic chains (green triangles, Figure 4b), can indicate that the backbone of the copolymer has been slightly altered. Considering the fact that a chain scission is a common negative phenomenon occurring during the laser ablation of polymers, also in the MAPLE process [18], it is reasonable to claim that in the obtained EVA coatings the chain scission also took place. Most likely, the formation of a free radical on one of the carbon atoms of the aliphatic main chain led to chain scission through disproportional termination; however, crosslinking cannot be absolutely excluded. The possible, expected reaction paths for aliphatic chain scission are shown in Figure 5. The creation of new double bonds and some alkyl terminal or pending groups (e.g., -CH_3_ or CH-(CH_3_)_2_) from one side can decrease the intensity of the band at 1465 cm^−1^ (due to the decreased amount of CH_2_), but from the other side, the proximity of the newly formed bands (due to the presence of new CH_3_ groups (typical region—1470–1450 cm^−1^) to the position of this band also can increase absorption.

The presence of new CH_3_ groups and C=C bonds in the EVA copolymer structure will consequently have an influence on the absorption of the band at 1371 cm^−1^ (orange circles), which was originally associated with the methylene group of the vinyl acetate group. Moreover, it cannot be concluded that the chain scission did not take place as the intensity of this band did not increase, because any change of the VAc group leading to cleavage of the methylene group may compensate it, which is actually shown in a further analysis.

Considering the changes within the VAc groups, a noticeable increase in the absorbance intensity was observed for the 1737 cm^−1^ band (black squares, Figure 4b). Although the peak maximum is typically associated with an ester group, the increase in the intensity of this band may be determined by the appearance of carbonyl groups (C=O) from another functional group. This is particularly the case since the spectra show a slight right-hand broadening (towards lower wavenumbers) of the peak base (Figure 3). Bands at lower wavenumbers are more typical for ketones, aldehydes, and carboxylic groups. A slight decrease in the intensity of the band at 1240 cm^−1^, and thus a lower amount of C-O bonds, is consistent with this interpretation and has also been evidenced in previous research concerning the PLD processing of EVA copolymer. We showed that there is a strong tendency for ketone formation [12], and it results in one single carbon-oxygen bond less per new ketone group. The possible reaction scheme of hydrogen elimination and ketone or carboxyl group formation is shown in Figure 5. It is important to realize that some of the new carbonyl groups can also originate from the oxidation of the aliphatic chain, even if it is less likely.

The defined changes of the chemical structure of the EVA copolymer are minor for all obtained coatings and show no dependency regarding the process conditions used. This emphasizes that, regardless of the process conditions used, for the system consisting of the EVA copolymer dissolved in chloroform, high structure preservation can be achieved. However, is has to be taken into account that the most susceptible part of the EVA macromolecule (according to statistical analysis) is the vinyl acetate block, which is consistent with the literature data which show that the carbonyl group of poly(lactide-co-glycolide) breaks during laser ablation due to the absorption of the high-energy photons [23].

The chemical structure analysis of the EVA coatings’ surfaces at the nanometer scale was performed using X-ray photoelectron spectroscopy (XPS). The advantage of the XPS technique is its much greater sensitivity; however, in the case of copolymers, it has to be taken into account that the chemical composition of the outer layer often differs from the bulk material due to the self-organization of macromolecules.

**Figure 6:**
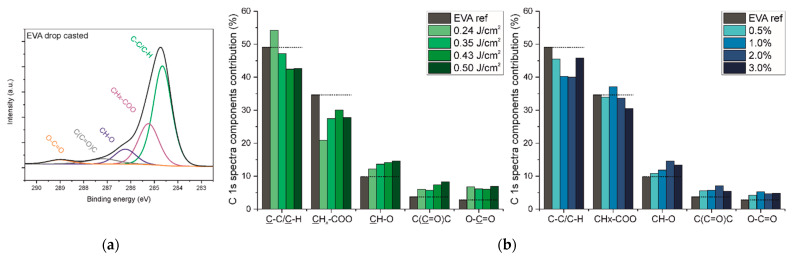
(**a**) High-resolution XPS C 1s spectra obtained for reference EVA coating. (**b**) Carbon components’ contributions from high-resolution XPS C 1s spectra deconvolution of two series of EVA coatings: left—green with variable laser fluences; right—blue with variable concentrations of the EVA solution. The individual bond contribution values of reference EVA coating were used for guidance in the analysis of MAPLE deposited coatings, shown as dotted line.

Two main atoms, carbon and oxygen, and traces of chlorine, originating from the solvent, were identified on the surface of the coatings. The elemental compositions of the surface of two series of EVA coatings are summarized in Table 2. The ratio of oxygen to carbon for the reference coating, i.e., 0.14, corresponds to the ratio of the EVA copolymer with 40 wt% of the VAc block [9]. The surfaces of all EVA coatings have similar chemical compositions to the reference coating surface; however, a small decrease in oxygen content was noticed for the series of coatings obtained for different laser fluences. Such differences on the surface composition for the EVA copolymer can result either from changes in the chemical structure or from the self-organization of the EVA blocks on the surface. The Et block has a lower surface energy than VAc; therefore, the enrichment of the surface by the Et block minimizes the surface tension favoring the self-organization [9,24]. This can be a reason for the lower O/C ratio on the surface. However, the analysis of high-resolution XPS C 1s spectra (discussed later, Figure 6) reveals that some changes in the chemical structure also took place.

The envelope of the XPS C 1s spectrum was deconvoluted into five components, as presented in Figure 6a for the reference EVA coating. The binding energy shifts of C 1s components are described in detail and reported in ref. [12] and presented here in Table 3.

The contribution of each carbon component of all EVA coatings’ surfaces is presented as two bar graphs in Figure 6b. Changes in the contribution of individual components from the C 1s spectra deconvolution for all investigated coatings have shown some common tendencies. In comparison with the reference EVA coating, the contributions of Comps. (1) and (2), assigned as carbon bonded with another carbon or hydrogen (C-C, C-H, 284.7 eV; CHx-CO, 285.2 eV), were slightly reduced in favor of the rest of the components. Comps. (3), (4), and (5) (286.2, 287.1, 289.1 eV) are associated with a carbon bonded with one atom of oxygen by single or double bond or with two atoms of oxygen. The increases in comps. (4) and (5) are in line with the above-mentioned mechanism underlying the formation of new ketone and carboxylic functional groups during the MAPLE process. An increase in comp. (3), denoted as carbon bonded with one oxygen atom by single bond, C-O, is in contrast to the bulk structure analysis (based on IR spectra), in which a slightly decreased amount of C-O bonds was defined. It might suggest that this particular change in chemical structure only occurs on the surface and probably only on the outer part of transported clusters. The literature indicates that the outer parts of transported molecules, e.g., nano/microparticles, undergo some structural changes due to local thermal effects through laser energy absorption by the particle [25]. Considering the morphology of EVA coatings (Figure 1 and Figure 2), i.e., higher roughness and bigger particles defined for coatings obtained with a higher laser fluence or higher concentration of solution, more pronounced structural changes for coatings 1%_EVA_0.43, 1%_EVA_0.50, 2%_EVA_0.50, and 3%_EVA_0.50 might be a result of significant thermal effects in the case of bigger clusters transported to the substrate.

The presence of chlorine in all surface coatings indicates either that chloroform molecules have been entrapped between macromolecules of EVA and, due to their interactions with macromolecules, could not evaporate or, more likely, that some chlorine radicals were created during the laser ablation and reacted with the macromolecules of the EVA copolymer. This undesirable consequence of using chloroform as a solvent for target in the MAPLE process is quite commonly known, but unfortunately, when the polymer is insoluble in other solvents, it is quite unavoidable. If chlorine is detected on the surface of a coating, such coatings should be utilized for medical applications with additional precautions. The fact that the higher amount of chlorine observed in the series with increasing fluence, achieved with a lower number of laser pulses, might suggest that this lower amount of chlorine (difficult to detect by infrared spectroscopy) can be the same across the entire coating. As already mentioned, the MAPLE process is time-dependent and since the concentration of the polymer in the target increases with time, at the beginning of the process (lower number of laser pulses), the amount of solvent is higher, and therefore the probability of creating chlorine radicals that are able to react with EVA is also higher. The chemical changes defined for the EVA macromolecule at some levels can perhaps be initiated by the chlorine radicals.

## 3. Materials and Methods

### 3.1. Materials

The EVA copolymer (ELVAX RESIN 40L-03; VAc content = 40 wt%, density = 0.967 g/cm^3^) was purchased from DuPont (Wilmington, DE, USA). Chloroform was purchased from Sigma Aldrich Co., Ltd. (St. Louis, MO, USA), and used without further purification. Double polished Si(100) pieces of 10 × 10 mm, transparent to IR, from Neyco Vacuum and Materials (Vanves, France) were ultrasonically cleaned in acetone and methanol and utilized as substrates. The reference sample was a drop-casted coating of EVA from solution prepared in chloroform placed on the Si substrate and dried.

### 3.2. MAPLE Process Parameters

Coating deposition was carried out in a NEOCERA system (Beltsville, MD, USA) for MAPLE processing using an Nd:YAG laser (model SL11-10, Continuum, Santa Clara, CA, USA) working at 266 nm, at fluences from 0.18 to 0.50 J/cm^2^ and 10 Hz pulse repetition rate. The laser fluences were set by keeping the spot area fixed (1.22 mm^2^) and changing the laser energy. The target-substrate distance was 3.7 cm. The base pressure in the chamber was between 10^−3^ and 10^−4^ mbar. Before the process, about 3000 pulses were used for the preablation step. During the laser irradiation, the target was evaporated and the polymer molecules were transported to two silicon substrates of ~1 cm^2^. The solutions used as targets for MAPLE process upon freezing were prepared in chloroform at concentrations of 0.5, 1, 2 or 3 EVA wt%. The solutions were frozen in liquid nitrogen (Linde Gaz România, B, Romania) to form target, placed in a vacuum chamber and subjected to laser irradiation.

### 3.3. Methods

The surface morphologies of the coatings were evaluated by optical microscopy observations using an Olympus BX51M microscope (Olympus Corporation, Tokyo, Japan) working in reflection mode, with magnification ranging from ×5 to ×100, as well as by atomic force microscope Park Systems, XE-100 model (Suwon-Si, Gyeonggi-Do, Korea). The AFM image area of 40 × 40 µm^2^ was obtained in non-contact mode using a silicon tip with a radius of less than 7 nm (nominal values) upon applying a constant force of 42 N/m, under a resonance frequency of 330 kHz. The thicknesses of the films were determined by the surface step height measurement using KLA-TENCOR P7 contact profilometer (Milpitas, CA, USA). The chemical structure was characterized using FTIR spectrometer (Jasco 6300, Easton, MD, USA) and XPS. For the IR analysis, 256 scans at a resolution of 4 cm^−1^ were carried out for each sample. The measurement was run in transmission mode. The quantitative analysis of FTIR spectra was performed by selecting reference band (at 2922 cm^−1^) and characteristic bands (1240, 1371, 1465 and 1737 cm^−1^) for spectrum of each coating and then by dividing the intensity of the particular characteristic band by the intensity of reference band. The statistical comparison was carried out based on Spencer et al.’s method [26] and one-way analysis of variance (ANOVA). The equations used and the numerical data are presented in Supplementary Materials. XPS measurements were performed using K-Alpha Thermo Scientific (ESCALAB™ XI+, East Grinstead, UK) spectrometer equipped with a monochromatic X-ray source AlKα 180 double focusing hemispherical analyzer. For the excitation of photoelectrons, X-ray radiation of an aluminum anode (AlKα, 1486.6 eV) was used. Survey spectra were recorded at pass energy of 50 eV in order to determine the surface elemental composition of the as-investigated materials. High-resolution spectra for C1s-binding energy regions were measured at pass energy of 20 eV in order to evaluate its binding states as a result of the investigated process parameters. Peak position was calibrated according to the standard C1s peak (284.8 eV). The spectra acquisition and data processing were performed by using the Avantage data software.

## 4. Conclusions

EVA copolymer coatings were successfully deposited using the MAPLE method. The quantitative analysis of the chemical structure showed minor changes both in the bulk and on the surfaces of the obtained coatings, mainly within the VAc blocks. It has been demonstrated that new ketone and carboxyl groups have arisen, and gentle changes within the aliphatic chain suggest some main chain scission. The alterations within the carbonyl group, i.e., laser susceptibility of the VAc block, are a consequence of the strong absorption of high-energy photons by C=O. This can lead to the general conclusion that regardless the process condition for polymers/copolymers a higher ratio of block with such heteroatoms as oxygen will increase the susceptibility of the macromolecule to chemical structure alteration. It can be also expected that the higher laser fluence used in the process will raise this negative effect.

Additionally, due to the nature of the interactions between macromolecules and/or solvent molecules, the EVA copolymer–chloroform system tends to form micro-/nano-clusters. Hence, the obtained coatings are characterized by considerable roughness, which varies depending on the laser fluence used or the concentration of the solution. The increases in polymer concentration and laser fluence lead to the deposition of thick coatings with high roughness. On the other hand, low concentrations and low fluence make it possible to obtain coatings that are much smoother, but at the same time with low thicknesses. The increase in thickness may be achieved in this case by increasing the process time/number of pulses. Therefore, the selection of deposition parameters should be primarily determined by the intended use of such coatings. For example, for applications requiring an increased adsorption of chemicals, such as in sensors, a high surface area development is desirable. In contrast, in, e.g., biological applications, where the low absorption of biomolecules is favorable, the roughness of the coating should be limited. Thus, the MAPLE method allows for the relatively simple control of this type of parameter and is therefore a promising tool for depositing thin films from polymer materials with complex structures.

However, it has to be highlighted that the recognition level of the generally accepted rule that the MAPLE process preserves the chemical structure of polymer material should strongly depend on the original polymer structure and the solvent used to prepare the solution. As we have shown, in the case of the EVA copolymer, made of blocks with different thermal-chemical resistances, solved in chloroform, very thorough structural studies have revealed that such changes can occur, and this fact could not be ignored in some specific applications.

## Figures and Tables

**Table 1:** Coatings’ names with the main process parameters.

Coating Name	EVA (wt%)	Fluence (J/cm^2^)	Pulse Number
1%_EVA_0.18	1	0.18	18,000
1%_EVA_0.24		0.24	
1%_EVA_0.35		0.35	
1%_EVA_0.43		0.43	
1%_EVA_0.50		0.50	
0.5%_EVA_0.50	0.5	0.50	36,000
1%_EVA_0.50 *	1.0		
2%_EVA_0.50	2.0		
3%_EVA_0.50	3.0		

**Table 2:** Chemical composition of the surface estimated based on XPS survey spectra.

Coating Name	C 1s	O1s	Cl 2p	O/C
EVA ref	87.49	12.51	-	0.14
1%_EVA_0.18	81.35	15.49	3.16	0.19
1%_EVA_0.24	87.08	9.75	3.16	0.11
1%_EVA_0.35	86.11	10.48	3.42	0.12
1%_EVA_0.43	84.85	9.31	5.84	0.11
1%_EVA_0.50	84.15	10.6	5.25	0.13
0.5%_EVA_0.50	87.89	10.30	1.81	0.12
1%_EVA_0.50 *	84.24	12.50	3.26	0.15
2%_EVA_0.50	86.40	12.44	1.16	0.14
3%_EVA_0.50	85.62	11.79	2.59	0.14

**Table 3:** Assignment of the C 1s components applied to the fitting procedure of high-resolution XPS C 1s spectra.

Component	Bond Assignment	Binding Energy (eV)
1	C-C, C-H,	284.7
2	CH_x_-CO	285.2
3	CH-O	286.2
4	C(C=O)C	287.1
5	O-C=O	289.1

## Data Availability

Data is contained within the article and Supplementary Material.

## Supplementary Materials

The following are available online at https://www.mdpi.com/article/10.3390/ijms222111686/s1.
