# Supplementary material for: Towards Understanding the Chemical Structure Modification of EVA Copolymer upon MAPLE Processing of Thin Films
Source: Int J Mol Sci. 2021 Oct 28;22(21):11686. doi: 10.3390/ijms222111686 (PMC8583955; doi:10.3390/ijms222111686)
Supplement: Supplementary file 1 [file ijms-22-11686-s001.zip › ijms-1409065-supplementary.pdf]

Supplementary Material

Towards Understanding the Chemical Structure Modification of EVA Copolymer upon MAPLE Processing of Thin Films

A quantitative statistical comparison of FTIR spectra was done basing on the Spencer et al. method [1] developed for absorbance comparison of FTIR spectra (specifically two bands).

The equation for an absorption band pair comparison:

$$\frac{A_{NS=1}(\nu_2)}{A_{NS=1}(\nu_1)} \pm \sigma_{\left[\frac{A_{NS=1}(\nu_2)}{A_{NS=1}(\nu_1)}\right]} \tag{1}$$

$$\sigma_{\left[\frac{A_{NS=1}(\nu_2)}{A_{NS=1}(\nu_1)}\right]} = \sqrt{N}\frac{A_{NS=1}(\nu_2)}{A_{NS=1}(\nu_1)} \times \left\{\left[\frac{1}{SNR^{A}_{NS=N}(\nu_2)}\right]\right\}^2 + \left\{\left[\frac{1}{SNR^{A}_{NS=N}(\nu_1)}\right]\right\}^2 \tag{2}$$

$$A_{NS=1}(\nu) = A_{NS=N}(\nu) \tag{3}$$

$$SNR^{A}_{NS=N}(\nu) = \frac{A_{NS=N}(\nu)}{\sigma^{A}_{NS=N}} \tag{4}$$

$A_{NS=1}(\nu)$—single-scan absorbance spectrum
$A_{NS=N}(\nu)$—a signal-averaged spectrum composed of N scans
$SNR^{A}_{NS=N}(\nu)$—signal-to-noise ratio
NS—the number of signal-averaged scans in a spectrum
$\sigma^{A}_{NS=N}$—average noise amplitude in an absorbance spectrum (determined from 1900–2000 cm^{-1})

	ν	$A_{NS=N}(\nu)$	$\sigma^{A}_{NS=N}$	$SNR^{A}_{NS=N}(\nu)$	$\frac{A_{NS=1}(\nu_2)}{A_{NS=1}(\nu_1)}$	$\sigma_{\left[\frac{A_{NS=1}(\nu_2)}{A_{NS=1}(\nu_1)}\right]}$
EVA REF	2922	1.231	0.0004	2930.952		
	1737	1.258	0.0004	2995.238		
					1.02	0.008
	2922	1.231	0.0004	2930.952		
	1240	1.093	0.0004	2602.381		
					0.89	0.007
	2922	1.231	0.0004	2930.952		
	1371	0.336	0.0004	800.000		
					0.27	0.006
	2922	1.231	0.0004	2930.952		
	1465	0.203	0.0004	483.333		
					0.16	0.006
0.24 J/cm^2	2922	0.028	0.00322	8.696		
	1737	0.029	0.00322	9.006		
					1.04	2.649
	2922	0.028	0.00322	8.696		
	1240	0.019	0.00322	5.901		
					0.68	2.224
	2922	0.028	0.00322	8.696		
	1371	-	0.00322	-		
					-	-

	2922	0.028	0.00322	8.696		
	1465	-	0.00322	-		
					-	-
0.35 J/cm^2	2922	0.147	0.00476	30.882		
	1737	0.177	0.00476	37.185		
					1.20	0.811
	2922	0.147	0.00476	30.882		
	1240	0.127	0.00476	26.681		
					0.86	0.685
	2922	0.147	0.00476	30.882		
	1371	0.042	0.00476	8.823		
					0.29	0.539
	2922	0.147	0.00476	30.882		
	1465	0.034	0.00476	7.1429		
					0.23	0.532
0.43 J/cm^2	2922	0.153	0.00262	58.397		
	1737	0.18	0.00262	68.702		
					1.18	0.423
	2922	0.153	0.00262	58.397		
	1240	0.133	0.00262	50.763		
					0.87	0.363
	2922	0.153	0.00262	58.397		
	1371	0.04	0.00262	15.267		
					0.26	0.283
	2922	0.153	0.00262	58.397		
	1465	0.021	0.00262	8.0153		
					0.14	0.277
0.50 J/cm^2	2922	0.147	0.00314	46.815		
	1737	0.162	0.00314	51.592		
					1.10	0.509
	2922	0.147	0.00314	46.815		
	1240	0.125	0.00314	39.809		
					0.85	0.449
	2922	0.147	0.00314	46.815		
	1371	0.039	0.00314	12.420		
					0.27	0.354
	2922	0.147	0.00314	46.815		
	1465	0.028	0.00314	8.9172		
					0.19	0.348

One-way ANOVA of band at 1737 cm^{-1}

ANOVA summary	
F	1.029
P value	0.3912
P value summary	ns
Significant diff. among means (P < 0.05)?	No
R squared	0.003217

95% Confidence Intervals (Dunnett)

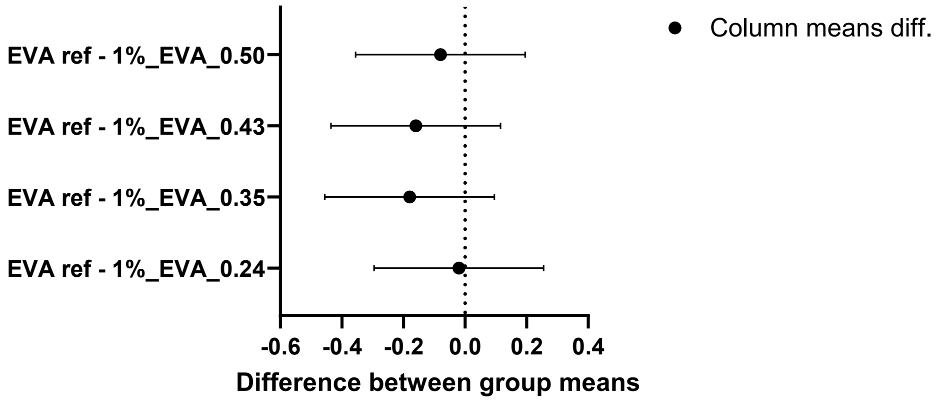

One-way ANOVA of band at 1240 cm^{-1}

ANOVA summary	
F	1.614
P value	0.1682
P value summary	ns
Significant diff. among means (P < 0.05)?	No
R squared	0.005039

95% Confidence Intervals (Dunnett)

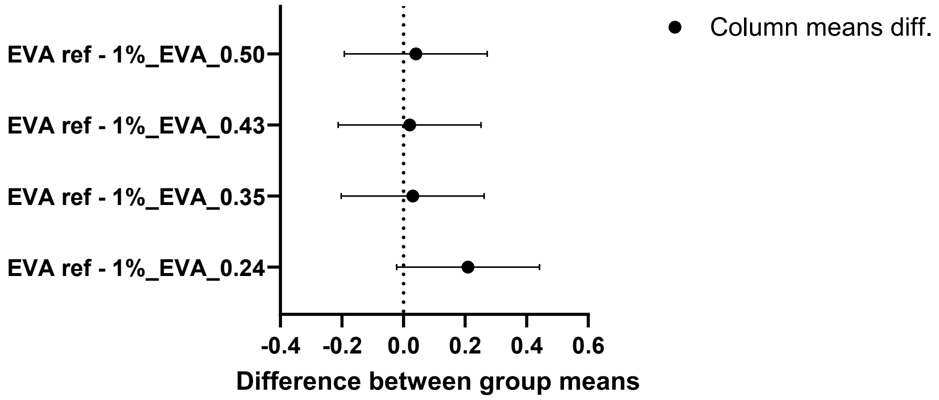

One-way ANOVA of band at 1371 cm^{-1}

ANOVA summary	
F	0.3269
P value	0.8059
P value summary	ns
Significant diff. among means (P < 0.05)?	No
R squared	0.0009606

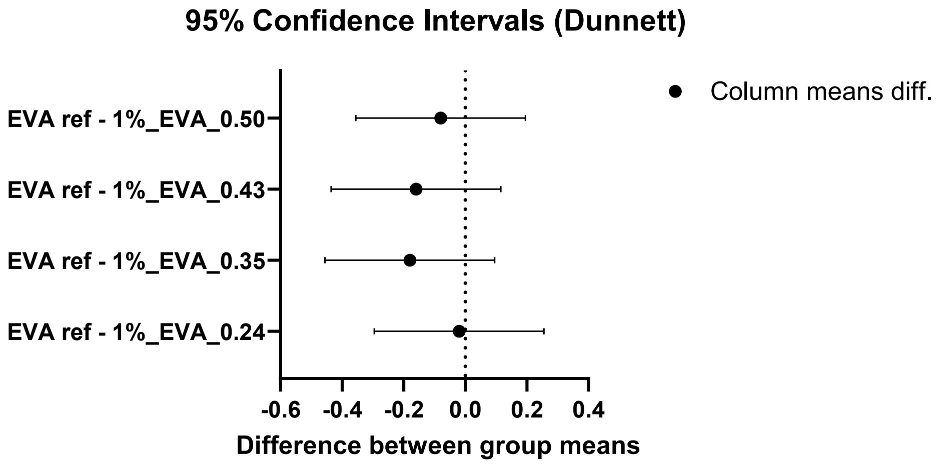

One-way ANOVA of band at 1465 cm^{-1}

ANOVA summary	
F	3.265
P value	0.0208
P value summary	*
Significant diff. among means (P < 0.05)?	Yes
R squared	0.009512

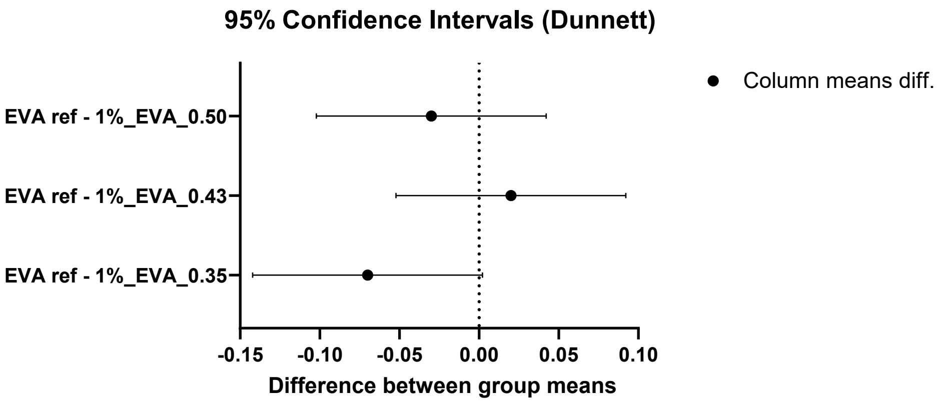

	ν	$A_{NS=N}(\nu)$	$\sigma^A_{NS=N}$	$SNR^A_{NS=N}(\nu)$	$\frac{A_{NS=1}(\nu_2)}{A_{NS=1}(\nu_1)}$	$\sigma_{\left[\frac{A_{NS=1}(\nu_2)}{A_{NS=1}(\nu_1)}\right]}$
EVA REF	2922	1.231	0.00042	2930.952		
	1737	1.258	0.00042	2995.238		
					**1.02**	**0.008**
	2922	1.231	0.00042	2930.952		
	1240	1.093	0.00042	2602.381		
					**0.89**	**0.007**
	2922	1.231	0.00042	2930.952		
	1371	0.336	0.00042	800		
					**0.27**	**0.006**
	2922	1.231	0.00042	2930.952		
	1465	0.203	0.00042	483.3333		
					**0.16**	**0.006**
0.5%_EVA_0.50	2922	0.267	0.00056	476.7857		
	1737	0.303	0.00056	541.0714		
					**1.13**	**0.051**
	2922	0.267	0.00056	476.7857		
	1240	0.21	0.00056	375		
					**0.79**	**0.043**
	2922	0.267	0.00056	476.7857		
	1371	0.08	0.00056	142.8571		
					**0.30**	**0.035**
	2922	0.267	0.00056	476.7857		
	1465	0.079	0.00056	141.0714		
					**0.30**	**0.035**
1%_EVA_0.50 *	2922	0.286	0.00248	115.3226		
	1737	0.313	0.00248	126.2097		
					**1.09**	**0.206**
	2922	0.286	0.00248	115.3226		
	1240	0.246	0.00248	99.19355		
					**0.86**	**0.183**
	2922	0.286	0.00248	115.3226		
	1371	0.077	0.00248	31.04839		
					**0.27**	**0.144**
	2922	0.286	0.00248	115.3226		
	1465	0.05	0.00248	20.16129		
					**0.17**	**0.141**
2%_EVA_0.50	2922	0.238	0.0055	43.27273		
	1737	0.273	0.0055	49.63636		
					**1.15**	**0.563**
	2922	0.238	0.0055	43.27273		
	1240	0.208	0.0055	37.81818		
					**0.87**	**0.491**
	2922	0.238	0.0055	43.27273		
	1371	0.064	0.0055	11.63636		
					**0.27**	**0.383**
	2922	0.238	0.0055	43.27273		
	1465	0.05	0.0055	9.090909		
					**0.21**	**0.378**
3%_EVA_0.50	2922	0.274	0.0035	78.28571		

	1737	0.289	0.0035	82.57143		
					**1.05**	**0.297**
	2922	0.274	0.0035	78.28571		
	1240	0.233	0.0035	66.57143		
					**0.85**	**0.268**
	2922	0.274	0.0035	78.28571		
	1371	0.078	0.0035	22.28571		
					**0.28**	**0.212**
	2922	0.274	0.0035	78.28571		
	1465	0.058	0.0035	16.57143		
	ν				**0.21**	**0.209**

One-way ANOVA of band at 1737 cm^{-1}

ANOVA summary	
F	8.301
P value	<0.0001
P value summary	****
Significant diff. among means (P < 0.05)?	Yes
R squared	0.02538

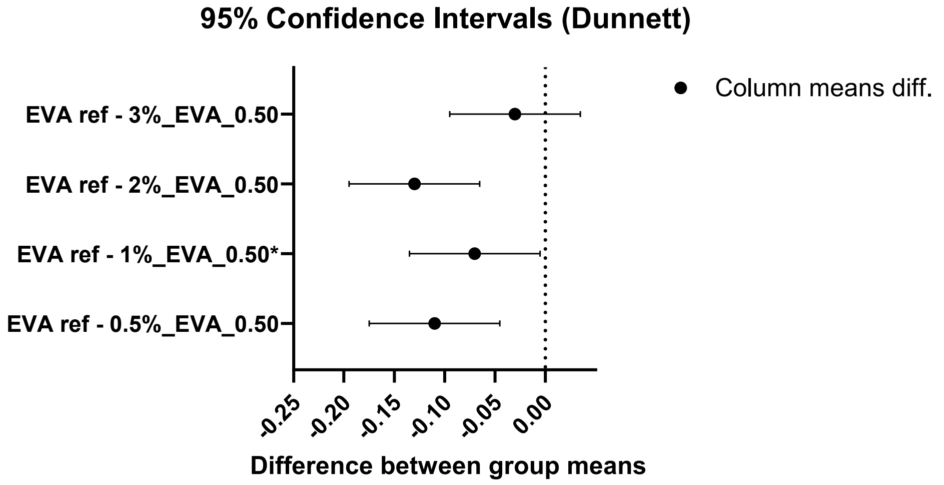

One-way ANOVA of band at 1240 cm^{-1}

ANOVA summary	
F	5.219
P value	0.0004
P value summary	***
Significant diff. among means (P < 0.05)?	Yes
R squared	0.01611

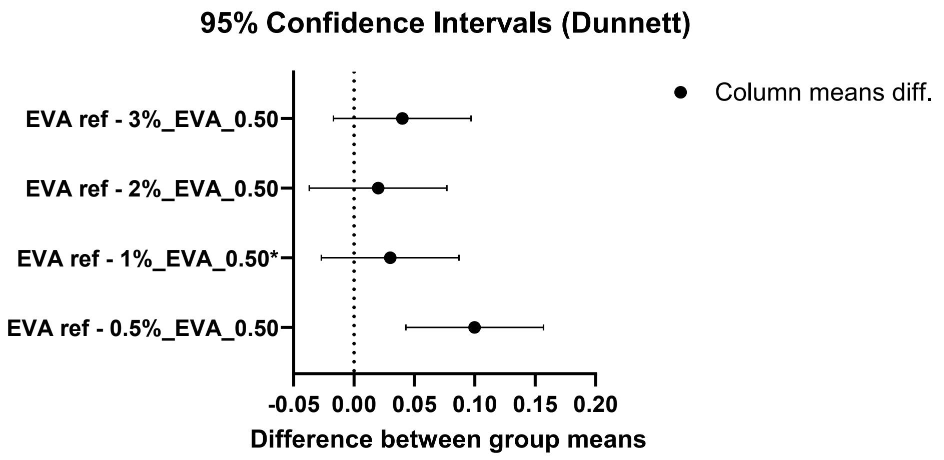

<u>One-way ANOVA of band at 1371 cm^{-1}</u>

ANOVA summary	
F	1.019
P value	0.3965
P value summary	ns
Significant diff. among means (P < 0.05)?	No
R squared	0.003185

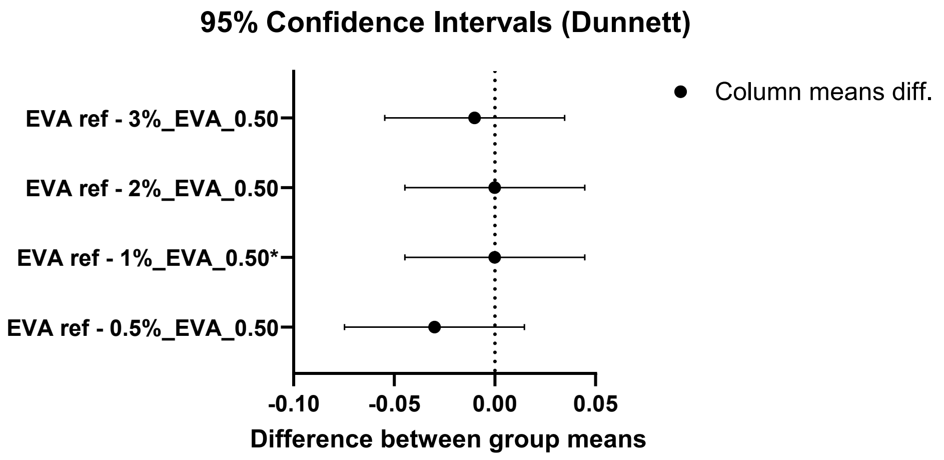

One-way ANOVA of band at 1465 cm⁻¹

ANOVA summary	
F	18.80
P value	<0.0001
P value summary	****
Significant diff. among means (P < 0.05)?	Yes
R squared	0.05568

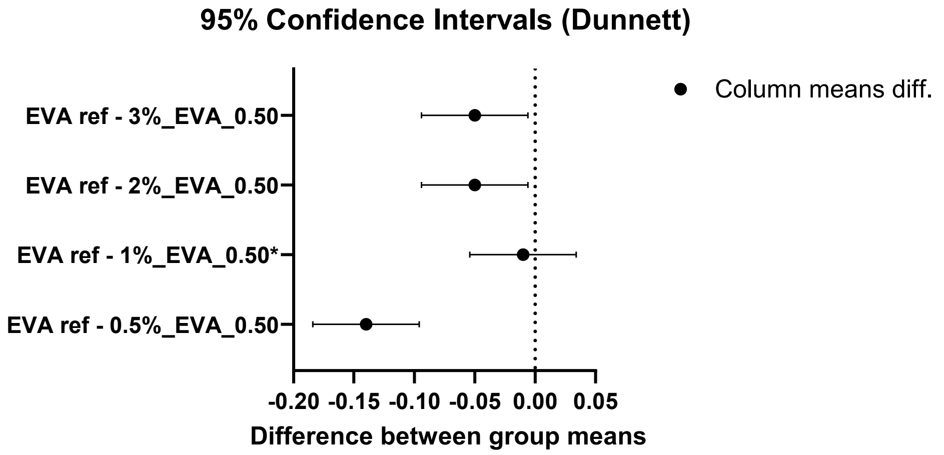

Reference

1. Spencer, R.G.; Calton, E.F.; Pleshko Camacho, N. Statistical comparison of Fourier transform infrared spectra. *J. Biomed. Opt.* **2006**, *11*, 064023, doi:10.1117/1.2393231.
